# Diagnostic Value of Bile Acids and Fibroblast Growth Factor 21 in Women with Polycystic Ovary Syndrome 

**DOI:** 10.1089/whr.2022.0060

**Published:** 2022-09-26

**Authors:** Jennie L. Yoost, Morgan Ruley, Kia Smith, Nalini Santanam, Holly A. Cyphert

**Affiliations:** ^1^ Department of Obstetrics and Gynecology, Marshall University, Huntington, West Virginia, USA.; ^2^ Department of Biological Sciences, Marshall University, Huntington, West Virginia, USA.; ^3^ Department of Biomedical Sciences, Joan C. Edwards School of Medicine, Marshall University, Huntington, West Virginia, USA.

**Keywords:** polycystic ovary syndrome, bile acids, hyocholic acid, obesity, FGF-21, FGF-19

## Abstract

**Objective:**

Polycystic ovary syndrome (PCOS) is a heterogeneous disorder characterized by a reduction in fertility and metabolic dysfunction. Unfortunately, due to a lack of clear presentation, it is often a long process of diagnosis. In this study, we investigated bile acids as potential biomarkers.

**Materials and Methods:**

Subjects were recruited and stratified into groups based on body mass index and PCOS status. Biometric data and plasma were acquired to understand bile acid profiles and related markers.

**Results:**

Taurocholic acid (TCA) and taurodeoxycholic acid were elevated in PCOS subjects with obesity in comparison to controls without PCOS. Fibroblast growth factor 21 (FGF-21), a metabolic regulator implemented in bile acid metabolism, was elevated in PCOS patients and was positively correlated with TCA changes.

**Conclusions:**

We present evidence suggesting that bile acids may be novel diagnostic targets in obese patients with PCOS while further studies need to delineate the interplay between FGF-21, bile acids, and testosterone in the early detection of PCOS.

## Introduction

Polycystic ovary syndrome (PCOS) is a disorder of metabolic and reproductive function and affects 6%–15% of women of reproductive age. Classic features include chronic anovulation, polycystic ovary morphology on ultrasound, and hyperandrogenism such as hirsutism, acne, or alopecia.^1^ Three guidelines that exist are overlapping, yet, both the Androgen Excess Society and the National Institutes of Health criteria require hyperandrogenism as a key diagnostic component.^2,3^ Polycystic ovaries alone can be seen in women with no other endocrine anomalies; therefore, hyperandrogenism remains a key aspect of this disease. It is known that women with PCOS are at increased risk for development of type 2 diabetes and metabolic syndrome (glucose intolerance, hypertension, dyslipidemia, and central obesity).^4^ It is therefore important to recognize this syndrome in adolescent women, as early intervention may prevent long-term sequelae.

Biomarkers have the potential to aid in earlier diagnosis of this complex disorder. It has been demonstrated that chronic inflammation may be a link between the metabolic disturbances in PCOS and hyperandrogenism.^5–7^ Many surrogate inflammatory markers have been studied among women with PCOS leading to a large heterogeneity of results.^8,9^ This is further compounded by obesity, as up to 64% of PCOS patients are obese.^10^ Obese PCOS subjects have increased androgens compared with normal weight PCOS subjects.^11^ Obese PCOS subjects also have increased androgens and fasting insulin levels compared with obese controls without PCOS.^1,6^ Despite an increased prevalence of obesity among women with PCOS, it is also known that normal weight PCOS patients also have an increased risk for metabolic disturbance.^12^ Hence, identification of biomarkers that are sensitive to metabolic derangements as seen in PCOS is required for early diagnosis.

Bile acids are involved in intestinal uptake of dietary lipids and fat-soluble vitamins in addition to acting as signaling molecules for cholesterol and lipid homeostasis.^13–15^ Bile acids also act as signaling molecules to regulate hepatic metabolism. Bile acids are first synthesized in the liver from cholesterol as primary bile acids, cholic acid, and chenodeoxycholic acid. Once synthesized, bile acids can be conjugated (taurinated/glycinated) and are subsequently stored in the gallbladder and are delivered to the gut after a meal. In the gut, bile acids can undergo bacterial deconjugation and dehydroxylation in the colon, yielding secondary bile acids (lithocholic acid, deoxycholic acid, ursodeoxycholic acid [UDCA]). Ninety-five percent of bile acids in the colon are recycled back to the liver *via* the enterhepatic circulation (only 5% are excreted into the feces).^13,15,16^

As several publications have eluded, bile acid levels and bile acid species composition (specifically changes in concentration of secondary and conjugated bile acids such as UDCA and glycoursodeoxycholic acid) are connected to insulin sensitivity following bariatric surgery.^14,17,18^ Following bariatric surgery, 80%–90% of insulin-insensitive patients experience a reversal in insulin sensitivity.^19–21^ This change is correlated with a change in bile acid pool size and species, suggesting a potential new feature and biomarker potential.^14^ As PCOS is associated with metabolic derangements independent of body mass index (BMI), we hypothesize that changes in certain bile acid species levels correlate with PCOS diagnostic criteria and hyperandrogenemia, making them a potential biomarker for diagnosis.

The goal of this study was to look at differences in bile acid species among women with known PCOS and controls, and to account for obesity by stratifying groups by BMI. In addition, we sought to understand the connection of bile acids with other factors associated with metabolic health, such as fibroblast growth factor-21 (FGF-21) and fibroblast growth factor 19 (FGF-19). As the incidence rate of PCOS continues to rise, it is vital to characterize the disease early, and biomarker analysis could potentially expedite this process, decreasing disease progression and improving patient outcomes.

## Methods

This is a cross-sectional study evaluating serum testosterone, insulin, metabolic regulators (FGF-19/FGF-21), and bile acids among young women with and without PCOS, stratified by BMI.

### Subjects

This study was conducted among women attending an academic OBGYN practice in Huntington, WV, during 2018/2019 before the COVID-19 global pandemic. Women aged 18–35 years were recruited into one of four subject groups: PCOS with obesity, PCOS without obesity, non-PCOS with obesity, and non-PCOS without obesity. Diagnostic criteria for PCOS included a history of chronic anovulation based on menstrual history, and clinical evidence of hyperandrogenism (acne, hirsutism, or alopecia).

Clinical or serum evidence of hyperandrogenism was included as key diagnostic criteria for study subjects as it is included in the most specific diagnostic guidelines. For the control non-PCOS subjects, inclusion criteria required no clinical evidence of hyperandrogenism (acne, hirsutism, or alopecia) and regular menstrual cycles. All subjects were at least 2 years from menarche. Obesity was defined by a BMI >30 kg/m^2^. This distinction was made to delineate alterations independent and dependent on weight as many of the markers evaluated are subject to change in an obese background.

Exclusion criteria included: (1) metformin use within past 3 months, (2) combination contraceptive use within past 3 months, (3) antibiotic use within 2 weeks of blood draw, (4) lactating, (5) pregnant within past 18 months, (6) known thyroid disease, (7) hyperprolactinemia, (8) liver disease, (9) other androgen disorders such as Cushing's disease, and (10) recent gastrointestinal illness within the past 2 weeks. Detailed clinical surveys were given to delineate medical history, age of menarche, family history specifically of PCOS, and cardiovascular and metabolic diseases such as type 2 diabetes. Once the subjects were recruited, their BMI was calculated using their weight and height. Informed consent was obtained from all study subjects. This study was approved by the Marshall Internal Review Board (1293149).

### Blood measurements

Recruited patients were subjected to venipuncture to collect blood for biochemical measurements following an overnight fast. Blood for bile acid analysis was immediately centrifuged for collection of plasma, which was then subsequently frozen at −80°C to maintain integrity. Bile acid plasma was sent to Michigan Metabolomics for analysis. Fasting serum insulin and testosterone levels were measured by Cabell Huntington Hospital Laboratories. FGF-19 and FGF-21 levels were assessed using ELISA (R&D) per the manufacturer's instructions.

### Bile acid measurements

Following plasma separation, samples were stored in the −80°C freezer before sending to the University of Michigan Metabolomics Core for analysis. Bile acids were separated and defined using a two-step solvent extraction and liquid chromatography–mass spectrometry separation by reverse-phase liquid chromatography and measurements by electrospray ionization triple quadruple multiple reaction monitoring methods (QQQ MRM).^22^

## Results

Twenty-seven subjects were recruited and consented for study participation. Subjects were placed into one of four groups based on their medical history with and without PCOS and their BMI (BMI >30 kg/m^2^, obese; BMI <30 kg/m^2^, nonobese). There were 15 subjects who met the clinical criteria for PCOS, and 12 control non-PCOS patients. These are further stratified by BMI in Table 1. There was no difference in age between groups. Subjects with PCOS (nonobese and obese combined) had an average age of menarche of 13.1 (standard deviation [SD] 2.56) compared with mean age of 12.2 (SD 1.13) among controls (*p* = 0.27) (Table 1).

**Table 1.** Biometric Data of Recruited Patients

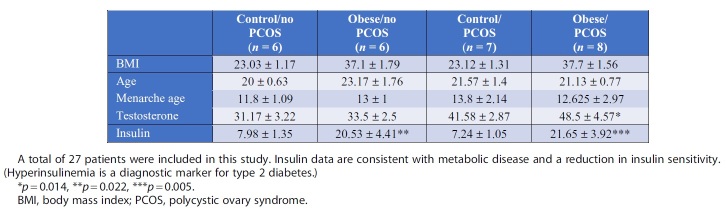

In regard to differentiation between nonobese and obese patients, the average age of menarche was 13.8 and 12.625, respectively (Table 1). Two PCOS subjects noted that they required hormonal therapy to induce menarche. PCOS subjects had a higher prevalence compared with controls of any first- or second-degree relative with PCOS (33.3% vs. 16.7%, *p* = 0.41), heart disease (73.3% vs. 25%, *p* = 0.021), or diabetes (80% vs. 25%, *p* = 0.007) (Table 2). PCOS subjects also had a higher reported incidence of menstrual cycle intervals greater than 90 days apart within the past year (73.3% vs. 0%, *p* = 0.001) (Table 2). As predicted with their history, subjects with PCOS had an increase in testosterone levels indicative of hyperandrogenism, independent of obesity (Table 1). Fasting insulin levels were also elevated in PCOS and non-PCOS subjects with obesity, highlighting possible insulin resistance (Table 1).

**Table 2.** Prevalence of a First- or Second-Degree Family Member with Polycystic Ovary Syndrome or Polycystic Ovary Syndrome-Related Conditions

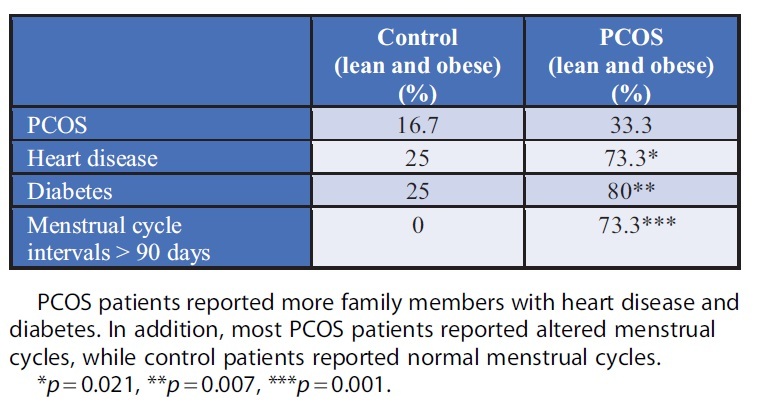

**FIG. 1.**
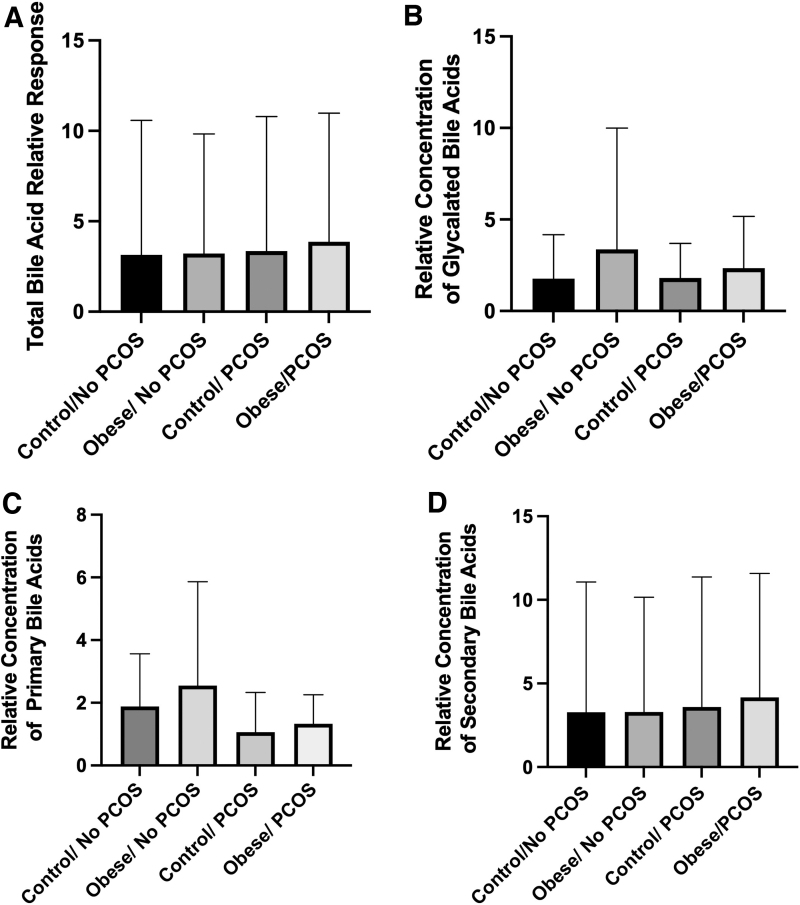
Overall bile acid levels or accumulation of glycine-conjugated bile acids are unchanged in PCOS. Subjects were subjected to a fasting blood draw to elucidate changes in bile acid species and accumulation. **(A)** Primary bile acids were unchanged. **(B)** Glycine-conjugated bile acids are unresponsive to PCOS metabolic disruption. **(C)** Primary bile acid (CDCA and CA) levels were not significantly altered in PCOS. **(D)** Secondary bile acids were unchanged in subjects with/without PCOS. CA, cholic acid; CDCA, chenodeoxycholic acid; PCOS, polycystic ovary syndrome.

**FIG. 2.**
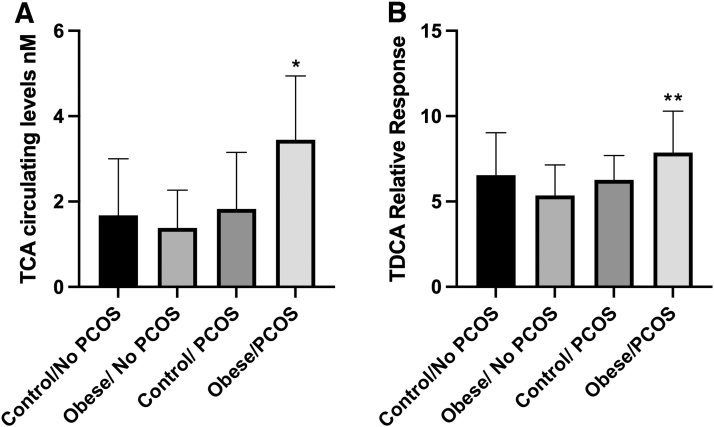
Subjects with PCOS have higher circulating levels of TCA and TCDCA compared with obese non-PCOS controls. **(A)** TCA and **(B)** TDCA are measured *via* relative concentration. *p = 0.04, **p = 0.05. TCA, taurocholic acid; TCDCA, taurochenodeoxycholic acid; TDCA, taurodeoxycholic acid.

Fasting bile acid levels in all the four groups of study participants were relatively unchanged (Table 3 and Fig. 1A). A more in-depth analysis of groups of bile acids also proved to be unrelated to PCOS as glycine-conjugated bile acids remained unaltered (Table 3 and Fig. 1B). Primary and secondary bile acids were also unaltered in patients with PCOS (Fig. 1C, D). Interestingly taurocholic acid (TCA) levels, a primary bile acid, increased significantly in PCOS patients with obesity compared with obese non-PCOS patients, reflecting a compounding change due to the metabolic state of the patient (Fig. 2A). In addition, taurodeoxycholic acid (TDCA) was only significantly elevated in obese PCOS patients compared with obese non-PCOS patients (Fig. 2B). In total, these data suggest that only some bile acids are altered in PCOS and that obesity with PCOS reflects a different bile acid expression pattern in comparison to PCOS patients who exhibit a normal BMI.

**Table 3.** Relative Response of Bile Acids Unaltered by Body Mass Index and Polycystic Ovary Syndrome Status

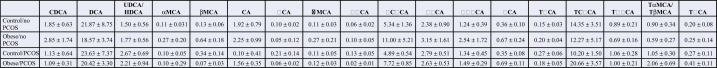

Data are shown as relative rate average ± standard error of the mean.

αMCA, alpha-muricholic acid; βMCA, beta-muricholic acid; ωMCA, omega-muricholic acid; CA, cholic acid; CDCA, chenodeoxycholic acid; DCA, deoxycholic acid; GCA, glycocholic acid; GCDCA, glycochenodeoxycholic acid; GDCA, glycodeoxycholic acid; GLCA, glycolithocholic acid; GUDCA, glycoursodeoxycholic acid; HCA, hyocholic acid; TCDCA, taurochenodeoxycholic acid; THCA, 3α,7α,12α-trihydroxycholestanoic acid; TLCA, taurolithocholic acid; TMCA, tauromuricholic acid; TUDCA, tauroursodeoxycholic acid; UDCA, ursodeoxycholic acid.

**FIG. 3.**
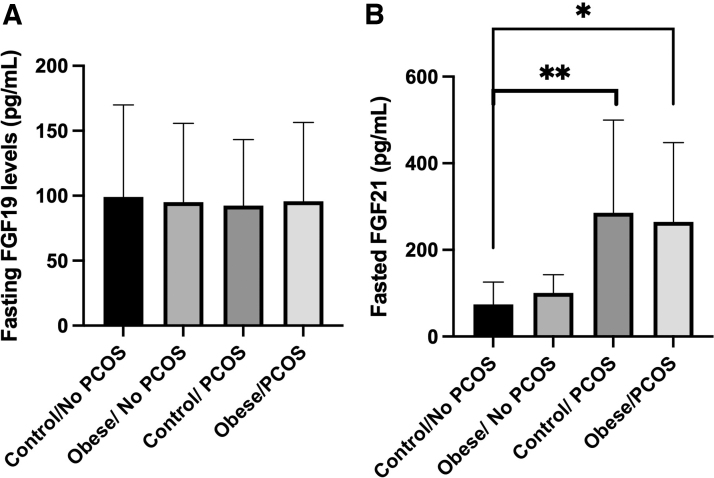
FGF-21, not FGF-19, is positively correlated with PCOS. **(A)** FGF-19 levels remain consistent across all the groups. **(B)** In both lean and obese backgrounds, FGF-21 levels are enhanced compared with non-PCOS subjects (*p = 0.0306, **p = 0.038). FGF-19, fibroblast growth factor 19; FGF-21, fibroblast growth factor 21.

We next evaluated molecular regulators and factors associated with bile acid metabolism. FGF-19 and FGF-21 are part of the fibroblast growth factor family of signaling molecules that are involved in regulating bile acid, glucose, and lipid metabolism.^23–25^ FGF-21 is a starved state hormone that augments and stages alterations in glucose metabolism to maintain homeostasis. Interestingly, FGF-21 levels are also augmented during metabolic diseases such as obesity and type 2 diabetes have increased concentrations.^23–25^ It has been previously reported that FGF-19 and FGF-21 levels are augmented in PCOS; however, there are also some reports that FGF-19 and FGF-21 levels are unchanged.^26–28^ In our population, FGF-19 levels were unchanged in the fasted state, in the normal weighted and obese background (Fig. 3A). In both weighted backgrounds, FGF-21 levels were significantly increased by 2.5- to 3-fold (Fig. 3B).

**FIG. 4.**
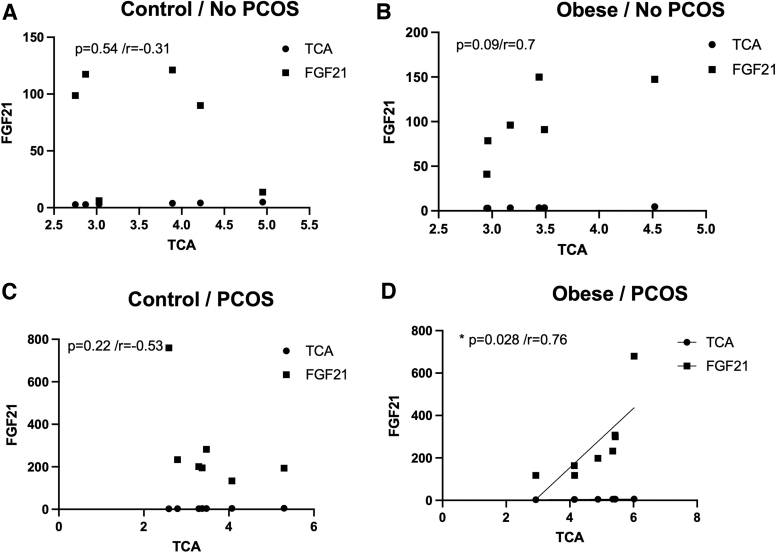
FGF-21 levels correlate with increased levels of TCA in obese PCOS patients. **(A–D)** Using Pearson's correlation analysis, fasting levels of TCA were positively correlated with higher levels of circulating FGF-21 only in patients with PCOS who were also obese **(D)**. (*p = 0.028/*r* = 0.76).

**FIG. 5.**
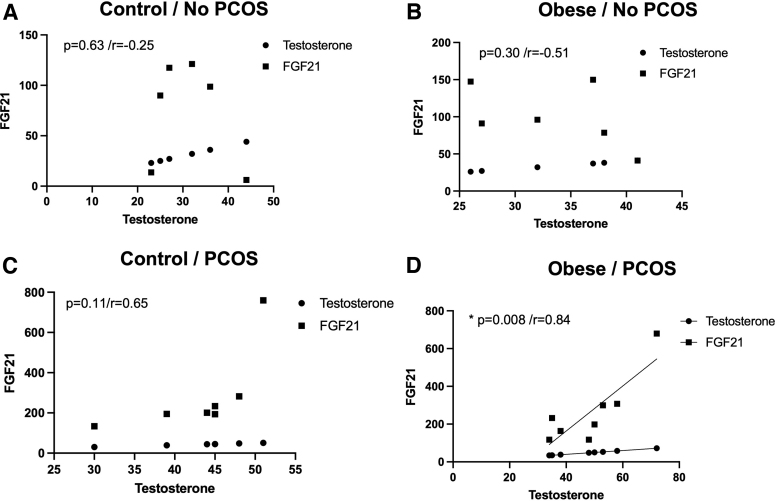
FGF-21 levels correlate with increased levels of testosterone. **(A–D)** Using Pearson's correlation analysis, fasting levels of testosterone were positively correlated with higher levels of circulating FGF-21 only in obese PCOS patients **(D)**. (*p = 0.008/*r* = 0.84).

PCOS obese patients with higher levels of FGF-21 also had higher levels of TCA, perhaps suggesting a regulatory role of FGF-21 in bile acid accumulation in PCOS (Fig. 4D). In addition, we also saw a correlation between higher FGF-21 levels with higher testosterone levels, again suggesting a possible interplay in obese patients with PCOS (Fig. 5D). We did not observe any correlation between TCA and FGF-21 or testosterone and FGF-21 in the control subjects (lean or obese) or in PCOS nonobese subjects (Figs. 4A–C and 5A–C). This suggests that obesity in PCOS has a different signaling profile in comparison to PCOS in a lean individual.

## Discussion

At the time of sample collection, this was thought to be the first study to elucidate bile acid species and pool in conjunction with other metabolic factors in PCOS patients. Only two bile acids, TCA and TDCA, were altered in PCOS patients compared with controls. Interestingly, TCA has been shown to increase FGF-21 levels in primary rat hepatocytes, perhaps showing a connection between bile acids and FGFs in the PCOS condition.^29^ Additionally, FGF-21, not FGF-19, was positively correlated with PCOS with and without obesity in our study. Previously, it has been noted that FGF-21 levels are increased in PCOS patients.^28,30^ In Olszanecka-Glinianowicz et al, it is observed that FGF-21 levels are elevated in PCOS patients, regardless of weight, compared with non-PCOS controls.^31^

In addition, FGF-21 levels are elevated in obese PCOS subjects in comparison to lean PCOS subjects, a result we did not see.^31^ This discrepancy in data could be a result of differences in the population or the period at which samples were collected regarding the subjects' menstrual cycle (we did not restrict blood draw to 3–5 days before menstrual cycle as was indicated in Olszanecka-Glinianowicz et al.) In addition, insulin levels were quite different between our data sets (*e.g.*, obese PCOS insulin levels in our study average 21.65 μIU/mL vs. 12.9 μIU/mL^31^ in their population), which could also account for differences in FGF-21 as insulin is positive regulator of hepatic FGF-21 levels.^29^ FGF-19 and FGF-21 are involved in metabolic balance; they are also implicated in lipid and bile acid metabolism.

FGF-21 has been explored as an antidiabetic hepatokine for the treatment of type 2 diabetes with somewhat promising results.^32^ FGF-21 has been shown to have tissue-specific effects, but ultimately pharmacological overexpression of FGF-21 recapitulates metabolic starvation, thus promoting fuel utilization and decrease in adiposity.^23^ Indeed, pharmacological overexpression beyond physiological levels could be a promising research goal to explore for the treatment of PCOS. Finally, during the course of our data collection and analysis period, other groups looked at bile acids, FGF-19 and FGF-21 levels in PCOS patients with varying recruitment criteria and conditions.^28,33,34^ FGF-19 levels have been published as decreased or unchanged in patients with PCOS.^27^ As our recruitment population was from an underserved rural area with high levels of diabetes and obesity, it is possible that distribution patterns of bile acids are affected heterogeneously depending on environmental queues.

Strengths of this study include stratification of PCOS subjects and controls by BMI, the inclusion of younger women, and the inclusion of both hormonal studies and potential biomarkers. Weaknesses include small sample size; however, this did allow for control of other variables such as the influence of medications, which could augment molecular pathways. We also examined bile acids in coordination with other metabolic changes. Therefore, showing correlation of bile acids with known diagnostic markers of PCOS further strengthened our study. PCOS is a heterogeneous disorder that often takes years to diagnose.

The goal of this study was to better define other signaling markers that could in fact be used to more efficiently diagnose PCOS. According to our data, there were not significant changes in bile acid total pool with only changes in two individual species, yet FGF-21 levels were consistently elevated among PCOS patients independent of obesity in coordination with TCA levels. This could be further explored as a dual marker for diagnosis. Future research will explore the use of these specific bile acid species and FGF-21 as early diagnostic markers of PCOS in adolescent women.

## Abbreviations Used

αMCA: alpha-muricholic acid
βMCA: beta-muricholic acid
ωMCA: omega-muricholic acid
BMI: body mass index
CA: cholic acid
CDCA: chenodeoxycholic acid
DCA: deoxycholic acid
FGF-19: fibroblast growth factor 19
FGF-21: fibroblast growth factor 21
GCA: glycocholic acid
GCDCA: glycochenodeoxycholic acid
GDCA: glycodeoxycholic acid
GLCA: glycolithocholic acid
GUDCA: glycoursodeoxycholic acid
HCA: hyocholic acid
PCOS: polycystic ovary syndrome
SD: standard deviation
TCA: taurocholic acid
TCDCA: taurochenodeoxycholic acid
TDCA: taurodeoxycholic acid
THCA: 3α,7α,12α-trihydroxycholestanoic acid
TLCA: taurolithocholic acid
TMCA: tauromuricholic acid
TUDCA: tauroursodeoxycholic acid
UDCA: ursodeoxycholic acid
